# Hemorrhagic herpes zoster duplex unilateralis in a patient taking clopidogrel

**DOI:** 10.1016/j.jdcr.2023.04.014

**Published:** 2023-04-26

**Authors:** Austinn C. Miller, Anthony Thompson, Alexzandra Mattia, Laurie A. Temiz, Susuana Adjei, Stephen K. Tyring

**Affiliations:** a Dermatology Associates of Tallahassee, Tallahassee, Florida; b Florida State University College of Medicine, Tallahassee, Florida; c Center for Clinical Studies, Webster, Texas; d Meharry Medical College, Nashville, Tennessee; e Department of Dermatology, University of Texas Health Science Center, Houston, Texas

**Keywords:** clopidogrel, coronary artery bypass graft, hemorrhagic herpes zoster, HHZ, multidermatomal

*To the Editor:* We read with interest the publication by Sowell et al^1^ on hemorrhagic herpes zoster (HHZ) in a contralateral, multidermatomal distribution in a patient taking rivaroxaban. To our knowledge, this was the first case reported to the literature implicating direct oral anticoagulants in the pathogenesis of this condition. Here, we present a case of HHZ in an ipsilateral, multidermatomal distribution in a patient who recently started clopidogrel.

A 75-year-old man receiving clopidogrel status–post distant coronary artery bypass graft presented with a vesicular rash. Two days prior, he noted simultaneous onset of dark vesicles on his cheek, buccal mucosa, lower abdomen, and back, consistent with a right-sided, ipsilateral distribution in the T10 and V3 dermatomes (Fig 1). Besides mild pain in the involved dermatomes, he denied any other symptoms. The patient was not vaccinated against varicella-zoster. Medical history was negative for HIV, malignancy, transplant, diabetes mellitus, systemic corticosteroids, and immunosuppressive drugs. The patient denied using any medications other than clopidogrel. No culture or PCR was performed based on a presumptive clinical diagnosis of HHZ duplex unilateralis. In support of the diagnosis, the patient had excellent clinical response to valacyclovir 1 g three times a day for 7 days. Clopidogrel was continued without alteration of dosage. The patient had no residual findings (ie, postherpetic neuralgia) at 3-week follow-up.

**Fig 1:**
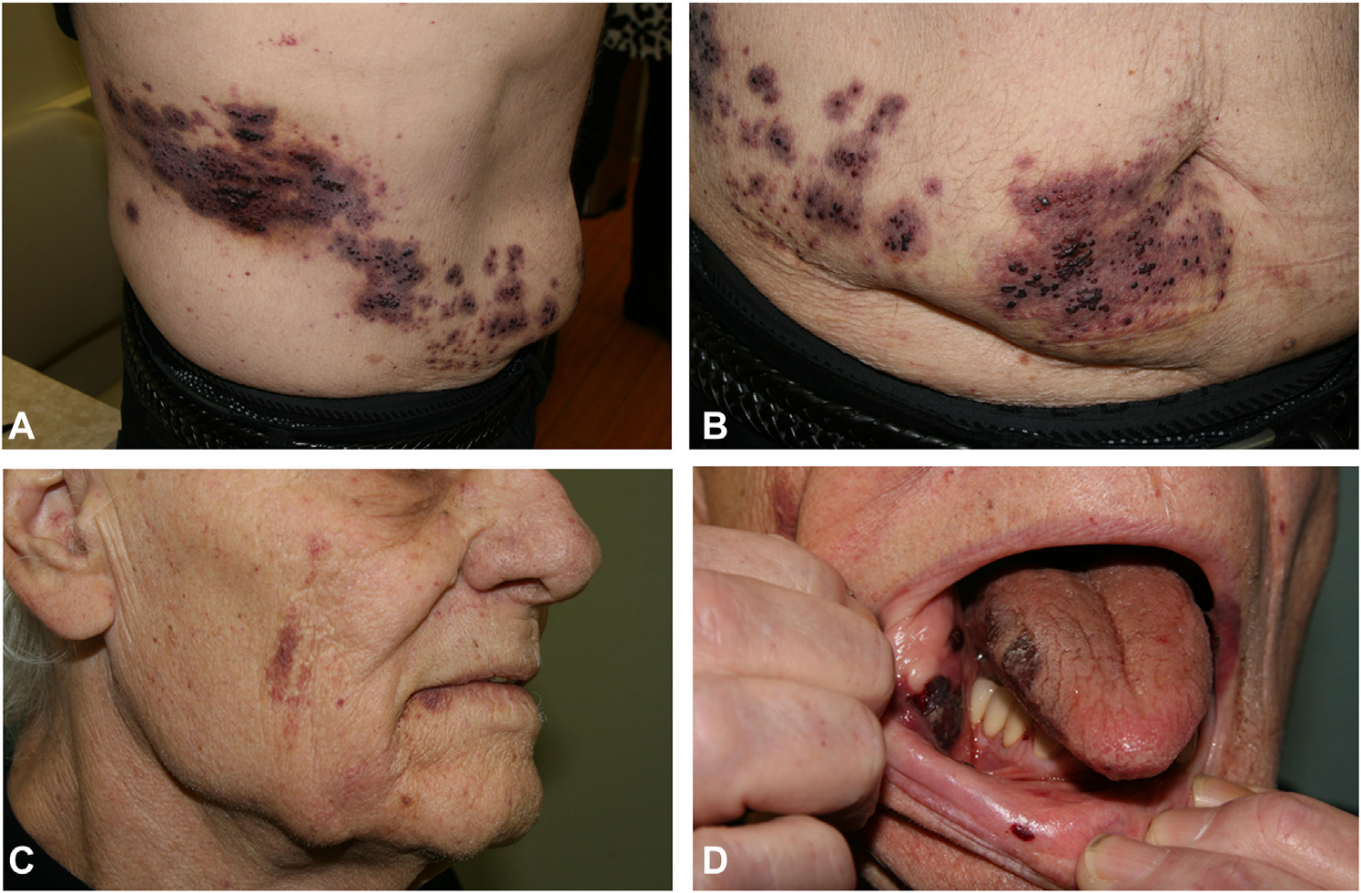
**A,** The lateral view of the right side of the T10 dermatome. **B**, The anterior view of the right side of the T10 dermatome. **C**, The lateral view of the right side of the V3 dermatome. **D**, The anterior view of the right side of the V3 dermatome.

Herpes zoster involving multiple noncontiguous dermatomes accounts for <0.1% of all herpes zoster cases.^2^ HHZ is also very rare and has been attributed to anticoagulant use in several cases.^1^^,^^3^ Both are commonly associated with immunodeficiency. Concomitant occurrence of both variants has only been reported once in a contralateral distribution involving C2, C3, V3, and L3 in a patient taking rivaroxaban.^1^ To our knowledge, there have only been 4 cases of HHZ secondary to clopidogrel reported in the literature.^4^ In all the cases, only 1 dermatome was involved, all individuals were immunocompetent, and the average at presentation was 70 years old (Table I).^3^^,^^4^ In 3 of 4 patients, clopidogrel was continued and treatment with valacyclovir or acyclovir was initiated with complete resolution of symptoms.

**Table I:** Summary of all cases of hemorrhagic herpes zoster associated with clopidogrel^3^^,^^4^

Previous cases	Age, y	Dermatome	Clopidogrel status	Immune status
Patient 1	49	T1	Discontinued	Immunocompetent
Patient 2	74	T1	Continued	Immunocompetent
Patient 3	78	L5	Continued	Immunocompetent
Patient 4	74	L5	Continued	Immunocompetent
Patient 5 (our case)	75	T10; V3	Continued	Immunocompetent

Our case corroborates Sowell et al^1^ and their suggestion that multidermatomal involvement should not deter providers from a diagnosis of herpes zoster. In this rare manifestation of a common disease, we reinforce clopidogrel as a potential offending agent in the pathogenesis of HHZ and demonstrate the first case of HHZ in a multiple, noncontiguous ipsilateral dermatomal distribution. In addition, to our knowledge, this is the first case describing intraoral involvement of HHZ. Despite HHZ demonstrating a predilection for immunocompromised individuals, this case supports previous literature describing the potential for HHZ in immunocompetent patients on anticoagulation.

## Conflicts of interest

None disclosed.
